# Supplementary material for: Exercise Training-Enhanced Lipolytic Potency to Catecholamine Depends on the Time of the Day
Source: Int J Mol Sci. 2020 Sep 21;21(18):6920. doi: 10.3390/ijms21186920 (PMC7554872; doi:10.3390/ijms21186920)
Supplement: Supplementary file 1 [file ijms-21-06920-s001.pdf]

Table S1. Exercise training program.

Weeks	Speeds (m/min)	Time (min.)	Incline
1st week	15	30	5°
2nd week	20	30	5°
3rd week	20	40	5°
4th week	25	40	5°
5th week	25	50	5°
6th week	27	60	5°
7th week	30	70	5°
8th week	30	80	5°
9th week	30	90	5°

Table S2. Primers sequences for quantitative real-time PCR.

Gene Name	Sense Primer (5′–3′)	Antisense Primer (5′–3′)
(rat)		
Bmal1	TTCATGAACCCGTGGACCAA	CCCTGGAATGCCTGGAACA
Per2	TCTCAGAGTTTGTGCGATGATTTG	CACTGGGTGAAGGTACGTTTGG
18S	AAGTTTCAGCACATCCTGCGAGTA	TTGGTGAGGTCAATGTCTGCTTTC
(mice)		
Bmal1	ACGACATAGGACACCTCGCAGA	CGGGTTCATGAAACTGAACCATC
Per2	ATCAGCCATGTTGCCGTGTC	CGTGCTCAGTGGCTGCTTTC
Atgl	CACTTTAGCTCCAAGGATGA	TGGTTCAGTAGGCCATTCCT
Hsl	GGCAGTGGTGTGTAACTAGGATTG	ATCCATGCTGTGTGAGAACGC
18S	TTCTGGCCAACGGTCTAGACAAC	CCAGTGGTCTTGGTGTGCTGA
